# Ward Atmosphere and Its Predictive Factors Among Psychiatric Nurses in Closed Psychiatric Wards: A Latent Profile Analysis

**DOI:** 10.1155/jonm/1251723

**Published:** 2026-08-29

**Authors:** Deyu Liu, Liping Zhao, Jianjian Wang, Linjing Zhang, Yuan Luo, Haiye Ran, Junyu Liu

**Affiliations:** ^1^ Xiangya School of Nursing, Central South University, Changsha, Hunan, China, csu.edu.cn; ^2^ The Second Xiangya Hospital, Central South University, Changsha, Hunan, China, csu.edu.cn; ^3^ School of Nursing, Capital Medical University, Beijing, China, ccmu.edu.cn

**Keywords:** closed psychiatric wards, latent profile analysis, physical environment, psychiatric nurses, ward atmosphere, workplace violence

## Abstract

**Introduction:**

The ward atmosphere in closed psychiatric wards significantly influences nurses’ well‐being and quality of care. However, the formation mechanisms of ward atmosphere perceptions remain underexplored, and previous research has largely overlooked individual heterogeneity in how nurses perceive different dimensions of the ward environment. This study aims to identify distinct profiles of ward atmosphere among psychiatric nurses in closed wards and examine the predictive effects of physical environmental factors and workplace violence on profile membership.

**Materials and methods:**

This quantitative, cross‐sectional study adopted mixed nonprobability sampling combining purposive and convenience sampling and collected data via online questionnaires. A total of 1037 psychiatric nurses from 34 hospitals in China completed self‐report questionnaires between July and August 2024. Ward atmosphere was measured using the Chinese‐version Essen Climate Evaluation Schema. Physical environment characteristics were collected using a self‐developed questionnaire. Workplace violence was measured using the Workplace Violence Scale. Latent profile analysis was conducted using Mplus 8.3 to identify distinct ward atmosphere profiles. Multinomial logistic regression (R3STEP) was used to examine predictors of profile membership. All respondents provided electronic informed consent, and the study was approved by the institutional ethics committee.

**Results:**

Three distinct profiles of ward atmosphere were identified: “Triple‐High group” (10.8%), “Safety‐Deficient group” (77.1%), and “Support‐Depleted group” (12.1%). Compared with nurses in the Triple‐High group, those in the Safety‐Deficient and Support‐Depleted groups reported poorer ventilation, smaller ward size, less adequate safety measures, more frequent workplace violence, and higher rates of permission for smartphone use. Greenery, video surveillance, tidiness, and larger ward size differentiated the Safety‐Deficient group from the Support‐Depleted group.

**Conclusion:**

Psychiatric nurses’ perceptions of ward atmosphere are heterogeneous, with the majority experiencing safety deficits. Both physical environmental features and exposure to workplace violence are important predictors of these perceptions. Hospital administrators should implement targeted modifications to the physical environment of the wards and reduce workplace violence to enhance the ward atmosphere.

**Implications for Nursing Management:**

Nursing managers should acknowledge the heterogeneity in nurses’ perceptions of ward atmosphere. Targeted improvements to ward physical environments—such as enlarging ward space, enhancing ventilation, and upgrading safety facilities—are recommended. Effective strategies should also be developed to mitigate workplace violence in closed psychiatric wards. Simple, low‐cost environmental adjustments may improve ward atmosphere, potentially alleviate psychological stress, and further contribute to nurse well‐being and retention.

## 1. Introduction

Nurses’ occupational health is intrinsically linked to the quality of their work environment, and poor working conditions have become a global occupational health challenge [1]. In psychiatric wards, particularly those that are closed, the inherently restricted environment intensifies patient–patient and patient–staff interactions, creating a unique and complex dynamic. At the same time, the unique characteristics of the patient population increase nurses’ exposure to critical incidents such as violence and suicide [2, 3]. This, in turn, creates a more pressing demand for a healthy and supportive work environment [4], which helps explain why the turnover rates are generally higher among psychiatric nurses than those in other departments [5]. Therefore, it is very necessary to study the work environment of psychiatric nurses, particularly those working in closed wards.

Ward atmosphere was developed specifically for psychiatric settings [6], which is used to measure the psychosocial perception of the working environment [7]. It reflects a dynamic interplay among architectural, organizational, staff, and patient characteristics [8]. The World Health Organization described the ward atmosphere as “the single most important factor in treatment efficacy” in inpatient mental health services [9], which is a key determinant of organizational health [10]. Schalast et al. [11] suggested that ward atmosphere could be conceptualized across three core dimensions: Therapeutic Hold, Patients’ Cohesion and Mutual Support, and Experienced Safety. These dimensions collectively capture the perceptions of both patients’ and staff’s feelings of safety, security, unity, and support within the clinical environment [11].

The nurses’ perception of ward atmosphere affects their health, well‐being, and the quality of nursing work. A positive ward atmosphere has been associated with beneficial outcomes for nurses, including lower burnout [12, 13], lower stress [14], increased work satisfaction [15], and working alliance [13]. Thus, it is essential to investigate how psychiatric nurses perceive the ward atmosphere and to identify its predictive factors, in order to foster healthier work environments and enhance nurse well‐being.

The ward atmosphere is the product of complex interactions among multiple factors, with its formation mechanism being multifaceted. Studies have shown that before and after moving to a new environment, ward atmosphere changes [16]. Unit layout may reduce negative outcomes, such as stress and fatigue for staff [17]; natural light could lead to optimal staff productivity [18]. Biophilic design, such as fresh air and greenery, could promote emotional, mental, and spiritual health [19]. Particularly in resource‐limited regions such as sub‐Saharan Africa, problems such as poor ventilation, overcrowding, and lack of greenery are more pronounced [20, 21], potentially exacerbating nurse burnout and high turnover, thereby compromising care quality [22]. As early as the 19th century, Nightingale’s landmark works *Notes on Nursing* and *Notes on Hospitals* pioneered research on the hospital physical environment. She systematically confirmed that core physical factors including cross‐ventilation, ward spatial layout, cleanliness, and natural light determine patient recovery outcomes and daily nursing quality, laying the foundational theoretical basis for subsequent research linking ward physical settings to ward atmosphere [23, 24].

Emergency events can impair nurses’ mental well‐being and job performance [25]. Such incidents may also worsen nurses’ perceptions of ward atmosphere. Among such incidents, workplace violence (WPV) is particularly prevalent in psychiatric wards and deserves special attention [26]. The latest evidence indicated that nearly half of the 1000 nurses had experienced at least one type of WPV in the past 12 months of employment [27]. In closed psychiatric wards, where patient movement is restricted and contact with nurses is intensive, the incidence of violence may be even higher, with a particularly strong impact on nurses’ psychological safety. As an emergency incident during work time, WPV profoundly alters psychological perceptions [28], including emotional states [29] and sense of security [30]. Therefore, it is worthwhile to investigate whether WPV influences nurses’ perception of the ward atmosphere. However, relatively few studies have examined whether WPV directly affects the perception of ward atmosphere. Moreover, current research primarily focuses on the outcomes of ward atmosphere [31–33], with limited attention paid to its predictors. Hence, this study aims to identify key predictive factors of ward atmosphere, with a specific focus on the physical environment and WPV.

Nevertheless, the majority of previous studies employed a variable‐centered approach to measure ward atmosphere [8, 13, 34, 35], which lacked consideration of the individual heterogeneity of different dimensions. Latent profile analysis (LPA), an individual‐centered approach, classifies individuals with similar response patterns into the same category based on their responses to external continuous variables. LPA is used in many fields such as psychology and psychiatry, and its classification accuracy and effectiveness are significantly higher than traditional classification [36].

This study aims to utilize LPA to determine psychiatric nurses’ ward atmosphere perceptions and investigate the predictive effects of the physical environment and WPV, with the goal of developing targeted strategies to improve the ward atmosphere and promote nurse well‐being in the future.

## 2. Method

### 2.1. Design and Setting

This study was a quantitative cross‐sectional design. From July to August 2024, questionnaires were distributed to members of the Mental and Psychological Care Alliance of the National Center for Disorders through the WeChat platform. Respondents filled out the questionnaires anonymously by scanning the QR code. The study was conducted in 34 hospitals across 19 provinces in China, including Hunan, Anhui, Guangdong, Jilin, Gansu, Hubei, Fujian, Shanxi, Hebei, Yunnan, Jiangxi, Zhejiang, Henan, Liaoning, Sichuan, Beijing, Xinjiang, Guangxi, and Inner Mongolia.

### 2.2. Respondents

This study applied a mixed nonprobability sampling combining purposive sampling and convenience sampling. Purposive sampling was used to screen hospitals with standardized closed psychiatric wards. After obtaining informed consent from the head nurses or directors of the selected hospitals, convenience sampling was then adopted to recruit on‐duty psychiatric nurses through the channels of the Mental and Psychological Nursing Alliance. All questionnaires were distributed via QR code on the alliance’s WeChat group and completed anonymously online.

A total of 1485 eligible nurses were invited to participate, and 1460 completed the questionnaire (98.32% return rate). After excluding 123 invalid questionnaires due to missing data, completion time of less than 1 min, or logical inconsistencies, e.g., reporting 1 year of work experience while holding a senior professional title, 1337 valid questionnaires remained. According to the purpose of the survey, the inclusion criteria were as follows: (a) volunteered to participate in this study and signed informed consent, (b) were on‐duty psychiatric nurses with more than 3 months of working experience, and (c) worked in closed psychiatric wards. The exclusion criteria were as follows: (a) standardized training nurses (a newly graduated nurse participating in mandatory structured training) and (b) refresher training nurses (an experienced nurse pursuing specialized skill enhancement through short‐term elective training). The 300 respondents worked in open psychiatric wards and were excluded because this study specifically focused on closed psychiatric wards. Finally, 1037 psychiatric nurses were included in the study. Previous studies have confirmed that the minimum sample size of LPA is 500 [37]. The sample size met the statistical requirements.

### 2.3. Measurement

#### 2.3.1. Demographic Characteristics and Physical Environment

The following demographic data were collected: age, gender, marital status, educational level, and monthly income. Additionally, occupational information, including professional titles, working hospital level, working time, weekly working time, and employment type, was also collected.

#### 2.3.2. Physical Environment

The physical environment questionnaire was developed based on a review of relevant literature [38, 39]. It comprised two parts with a total of 12 items. The factual items, including the presence of greenery and video surveillance in the ward, permission for smartphone use, and smoking, were reported by the ward managers of the 34 participating hospitals through the nursing alliance network. The perceived items assessed eight dimensions of the ward environment: ward size, tidiness, ventilation, lighting, sounds, thermal comfort, adequacy of safety measures, and adequacy of privacy protection. These perceived items were self‐rated by respondents on a 10‐point numeric rating scale from 1 (*very poor*) to 10 (*very good*), with higher scores indicating better perceived environmental quality. A pretest was conducted among 35 frontline psychiatric nurses before formal investigation. Based on pretest feedback, we revised ambiguous item wording to optimize questionnaire quality.

#### 2.3.3. Ward Atmosphere

The Chinese version of the Essen Climate Evaluation Schema (EssenCES) by Schalast et al. [11] was used in this study, which was translated, cross‐culturally adapted, and psychometrically validated by our research team in a previously published study [40]. The EssenCES is a 15‐item questionnaire designed to assess the social and therapeutic atmosphere in psychiatric wards in three dimensions: patient cohesion and mutual support, experienced safety, and therapeutic hold. The respondents rated each item on a 5‐point Likert scale, with options ranging from 0 (*not at all*) to 4 (*very much*). Total scores are derived by summing item responses, yielding a composite range of 0–60. High scores on the EssenCES suggest a positive ward atmosphere. In this study, the internal consistency coefficient of the scale was found to be 0.784.

#### 2.3.4. WPV

The scale was developed by the Chinese scholar Wang [41] to assess the frequency of WPV in the past year. It comprises 5 items utilizing a 4‐point Likert‐type response format, with options ordered hierarchically by incident frequency. The score follows an ordinal system: 0 points for no violence, 1 point for 1 incident, 2 points for 2–3 incidents, and 3 points for ≥ 4 incidents. The total scale score is calculated by summing the item‐level scores (range: 0–15). A higher total score indicates a greater frequency of WPV. The scale also categorizes the frequency of WPV as follows: zero frequency (0 points), low frequency (1–5 points), medium frequency (6–10 points), and high frequency (11–15 points). In this study, Cronbach’s *α* for the WPV scale was 0.842.

### 2.4. Data Analysis

Data analysis was conducted using SPSS 29.0 and Mplus 8.3. SPSS29.0 software was used for statistical description and single‐factor analysis. Mplus 8.3 was used to explore the latent profiles of ward atmosphere among psychiatric nurses.

The data followed an approximately normal distribution. The measurement data are expressed as the means ± standard deviations (M ± SD), and the count data are expressed as frequencies and percentages.

Prior to statistical analysis, a common method deviation was performed with the Harman single‐factor test. The results revealed that the unrotated first factor explained 35.14% of the total variance, falling short of the 40% threshold [42]. Seven statistics were used to determine the number of latent profiles: Akaike information criterion (AIC), Bayesian information criterion (BIC), adjusted BIC (aBIC), Lo–Mendell–Rubin likelihood ratio test (LMRT), bootstrap likelihood ratio test (BLRT), entropy, and minimum class size. The smaller the AIC, BIC, and aBIC, the better the model fit [43]. LMR and BLRT were used to compare the fitting differences between k‐1 and *k* category models. Entropy was used to evaluate the accuracy of the classification, and its value ranged from 0 to 1, with the closer it was to 1, indicating that the classification was more accurate. Researchers also recommended that the smallest class should comprise at least 5% of the total sample [44]. Subsequently, the three‐step approach (R3step) of Mplus was used to model the predictors of profile membership. This command enables a series of multinomial logistic regressions that assess whether an increase in an independent variable results in a higher probability of a person belonging to one profile over another.

### 2.5. Ethics Approval and Consent to Participate

Ethical approval for the study was granted by the Ethics Committee of the Second Xiangya Hospital of Central South University (2024‐060). This research was performed in accordance with the Declaration of Helsinki. All respondents provided electronic informed consent by clicking a consent button before accessing the online questionnaire, and the study protocol was approved by the Institutional Review Board (IRB). Personal data were anonymized to ensure confidentiality.

## 3. Result

### 3.1. Demographic Data

The samples were collected from Hunan, Anhui, Guangdong, Jilin, Gansu, Hubei, Fujian, Shanxi, Hebei, Yunnan, Jiangxi, Zhejiang, Henan, Liaoning, Sichuan, Beijing, Xinjiang, Guangxi, and Inner Mongolia in China. Among the 1037 psychiatric nurses, there were 857 female (82.6%). Their average age and nursing working time were 34.85 ± 7.82 and 12.66 ± 8.11 years, respectively. The detailed demographic information is presented in Table 1.

**TABLE 1:** Sample demographic characteristics (*N* = 1037).

Variables	*N*	%	WA (M ± SD)
*Gender*			
Male	180	17.4	35.31 ± 6.73
Female	857	82.6	36.94 ± 6.95

*Educational level*			
Below the undergraduate	300	28.9	38.45 ± 9.16
Undergraduate	723	69.7	36.23 ± 6.71
Master and above	14	1.4	35.07 ± 6.01

*Marital status*			
Single	226	21.8	37.79 ± 6.99
Married	780	75.2	36.27 ± 6.89
Divorced/	30	2.9	38.20 ± 7.45
Widowed	1	0.1	35.00

*Monthly income(Yuan)*			
< 3000	89	8.6	36.42 ± 7.10
3000–5000	350	33.8	36.67 ± 7.23
5001–10,000	496	47.8	36.81 ± 6.88
10,001–20,000	99	9.5	35.97 ± 6.07
> 20,000	3	0.3	38.00 ± 2.00

*Professional titles*			
Primary title	548	52.8	37.58 ± 7.24
Intermediate title	411	39.6	35.42 ± 6.48
Senior title	78	7.5	36.39 ± 6.05

*Working hospital level*			
Primary‐level	9	0.9	31.88 ± 7.18
Secondary‐level	357	34.4	35.75 ± 6.30
Tertiary‐level	671	64.7	37.20 ± 7.18

*Employment form*			
In‐staff	367	35.4	36.35 ± 6.81
Off‐staff	670	64.6	36.82 ± 7.00

*Note*: M=mean. Professional title: Primary title, including nurses and nurse practitioners. Intermediate title included nurses‐in‐charge. The senior title included cochief nurses and chief nurses. Working hospital level: Primary‐level hospitals are community‐based healthcare institutions that directly serve local residents. Secondary‐level hospitals are regional medical centers that provide services across multiple communities. Tertiary‐level hospitals are large‐scale institutions offering specialized care across provinces, municipalities, and even nationwide. Employment form: In‐staff refers to nurses who hold a permanent establishment position within the public healthcare system. Off‐staff refers to nurses employed on a contractual or temporary basis without bianzhi status, which typically denotes permanent employment with pension benefits and job security in Chinese public institutions.

Abbreviations: SD, standard deviation; WA, ward atmosphere.

### 3.2. Identification of Ward Atmosphere Profiles

We conducted an LPA based on the scores of psychiatric nurses in the three dimensions of the ward atmosphere. To identify the best model, five models were separately estimated in this study, and Table 2 displayed the fit statistics results of each model. As the number of model categories increased from 1 to 5, the AIC, BIC, and aBIC values continued to decrease in the 1 to 4 models, and the 5 models were greater than the 4 models. Among the 5 models, the entropy was the highest in the 3 groups. However, there was no statistical significance between LMR and BLRT in the 4 models, which means that the 4 models are not more fitting than the 3 models. Therefore, the model with 3 profiles was finally chosen as the best potential profile model for this study. Table 3 shows the attribution probability matrix for the 3 potential profiles. The average probability of attribution of each class to its corresponding potential profile ranged from 90.7% to 94.5% (all > 90%), indicating that the results of the model for the three potential profiles in this study were plausible.

**TABLE 2:** Model fit indices for profile solutions.

Number	AIC	BIC	aBIC	LMRT (P)	BLRT	Entropy	Minimum class %
1	18,043.174	18,073.369	18,054.312				
2	17,621.370	17,671.696	17,639.933	0.0003	0.0004	0.828	165 (14.5%)
3	**15,825.271**	**15,894.488**	**15,850.023**	**<** **0.001**	**<** **0.001**	**0.843**	**122 (11.8%)**
4	15,726.696	15,815.689	15,758.519	0.1941	0.2022	0.739	77 (7.5%)
5	15,838.571	15,947.341	15,877.466	0.5224	0.5156	0.821	7 (0.6%)

*Note:* Bold values denote the selected optimal model (3‐class solution) based on lower AIC/BIC/aBIC, significant LMRT and BLRT (^∗^ *p* ^∗^ < 0.001), entropy = 0.843, and meaningful smallest class proportion (11.8%).

**TABLE 3:** Attribution probabilities for each latent profile.

Class	Profile 1	Profile 2	Profile 3
1	**0.945**	0.022	0.033
2	0.093	**0.907**	0.000
3	0.089	0.000	**0.911**

*Note:* Bold diagonal values are average posterior probabilities of correct classification for each latent profile.

**FIGURE 1:**
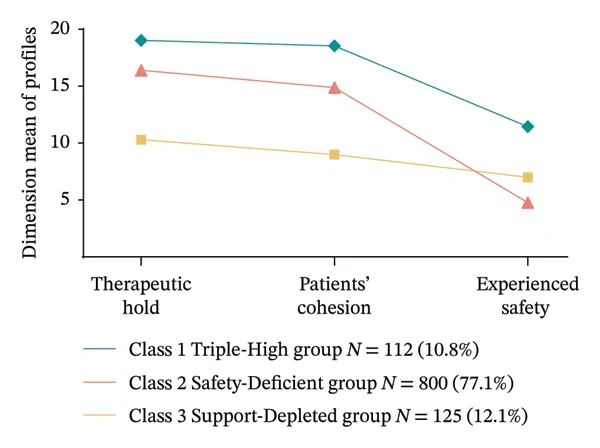
Mean scores of three dimensions of ward atmosphere.

Figure 1 reflected the distribution of each dimension of ward atmosphere in the 3‐profile model of ward atmosphere. The three potential profiles of ward atmosphere among psychiatric nurses scored differently and showed different characteristics. There were 112 psychiatric nurses (10.8%) with the highest score of each dimension in Profile 1. There were 800 psychiatric nurses (77.1%) with median score of Therapeutic Hold and patients’ Cohesion and the lowest score of Experienced Safety in Profile 2. There were 125 psychiatric nurses (12.1%) with the lowest score of Therapeutic Hold and patients’ Cohesion but the median score of Experienced Safety in Profile 3. Thus, Profiles 1–3 were named as “Triple‐High group,” “Safety‐Deficient group,” and “Support‐Depleted group,” respectively.

### 3.3. Predictors of Latent Profile Membership

**FIGURE 2:**
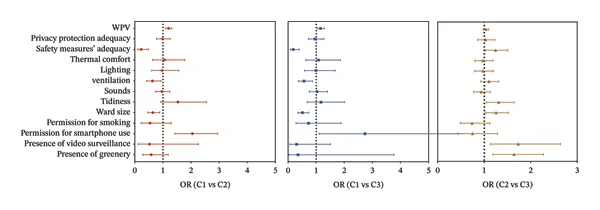
OR of predictors of latent profiles. Notes: The maximum value of the OR in the figure is displayed up to 5. Values exceeding this limit are not shown in the figure.

**FIGURE 3:**
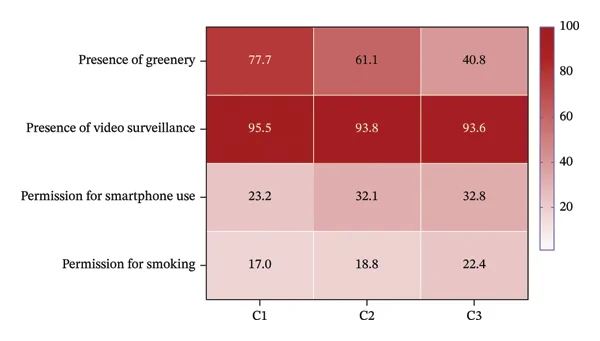
The distribution of predictor factors in each Profile 1. Notes: Draw a red graph showing the proportion of wards with greenery and video surveillance, where mobile phone usage is permitted and smoking is allowed.

**FIGURE 4:**
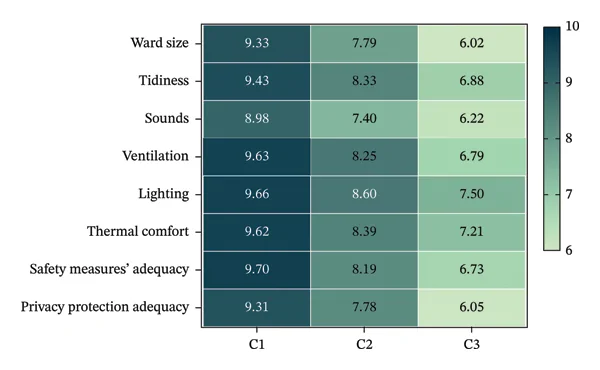
The distribution of predictor factors in each Profile 2. Notes: Draw a green graph showing the mean score of perception of ward environment (ward size, tidiness, sounds, ventilation, lighting, thermal comfort, safety measures’ adequacy, privacy protection adequacy).

Table 4 and Figure 2 presented the R3STEP results of predictive factors of ward atmosphere profile membership. Using the “Triple‐High group” profile as the reference group, nurses in “Safety‐Deficient group” profile and “Support‐Depleted group” profile achieved worse ventilation, fewer safety measures, smaller ward size, and encountered more WPV. Meanwhile, psychiatric nurses working with permission for smartphone use are more capable of perceiving the ward atmosphere of “Safety‐Deficient group” profile and “Support‐Depleted group” profile. Compared with the “Support‐Depleted group” profile, the “Safety‐Deficient group” profile was more likely to have greenery, video surveillance, bigger size, better tidiness, and more adequate safety measures. The heatmaps in Figures 3 and 4 clearly illustrate the distinct distribution of each factor across the three profiles.

**TABLE 4:** Three‐step results of predictors of latent profiles.

	C2 vs C1			C3 vs C1			C2 vs C3		
	Reference: C1			Reference: C1			Reference: C3		
	β	OR	95% CIOR	β	OR	95% CIOR	β	OR	95% CIOR
Presence of greenery	−0.538	0.584	0.286 ∼ 1.192	−1.033	0.356	0.034 ∼ 3.770	**0.496**	1.642**^∗^**	1.184 ∼ 2.277
Presence of video surveillance	−0.640	0.527	0.123 ∼ 2.267	−1.189	0.304	0.061 ∼ 1.511	**0.549**	1.732**^∗^**	1.134 ∼ 2.643
Permission for smartphone use	**0.718**	2.050**^∗∗^**	1.424 ∼ 2.953	**1.007**	2.738**^∗∗∗^**	1.107 ∼ 6.764	−0.289	0.749	0.435 ∼ 1.289
Permission for smoking	−0.616	0.540	0.225 ∼ 1.295	−0.313	0.731	0.284 ∼ 1.882	−0.303	0.739	0.485 ∼ 1.125
Perception of ward environment									
Ward size	**−0.445**	0.641**^∗∗^**	0.462 ∼ 0.889	**−0.673**	0.510**^∗∗∗^**	0.353 ∼ 0.737	**0.228**	1.256**^∗^**	1.036 ∼ 1.522
Tidiness	0.429	1.536	0.925 ∼ 2.551	0.158	1.171	0.683 ∼ 2.008	**0.271**	1.311**^∗^**	1.046 ∼ 1.644
Sounds	−0.036	0.964	0.747 ∼ 1.247	0.029	1.030	0.754 ∼ 1.406	−0.066	0.936	0.774 ∼ 1.132
Ventilation	**−0.467**	0.627**^∗∗^**	0.420 ∼ 0.935	**−0.566**	0.568**^∗∗∗^**	0.370 ∼ 0.871	0.099	1.104	0.929 ∼ 1.312
Lighting	−0.031	0.970	0.599 ∼ 1.569	−0.006	0.994	0.592 ∼ 1.670	−0.025	0.975	0.797 ∼ 1.193
Thermal comfort	0.058	1.059	0.631 ∼ 1.780	0.084	1.087	0.635 ∼ 1.864	−0.026	0.974	0.799 ∼ 1.188
Safety measures’ adequacy	**−1.459**	0.232**^∗∗∗^**	0.111 ∼ 0.486	**−1.680**	0.186**^∗∗∗^**	0.088 ∼ 0.394	**0.221**	1.247**^∗^**	1.031 ∼ 1.509
Privacy protection adequacy	−0.011	0.989	0.777 ∼ 1.259	−0.041	0.960	0.724 ∼ 1.273	0.030	1.030	0.864 ∼ 1.229
WPV	**0.183**	1.201**^∗∗∗^**	1.095 ∼ 1.318	**0.147**	1.158**^∗∗^**	1.042 ∼ 1.288	0.036	1.037	0.979 ∼ 1.097

*Note*: All binary predictors were coded as 1 = yes and 0 = no and treated as continuous variables in the R3STEP analysis. Values (β) in the table are estimates through the R3STEP logistic regression analyses using Mplus. Positive values (β) indicate that the antecedent makes an individual more likely to be classified into the first latent profile than the second latent profile; negative values (β) indicate the opposite. Bold values indicate statistically significant odds ratios (OR) and corresponding *β* coefficients (^∗^ *p* ^∗^ < 0.05).

^∗^ *p* < 0.05.

^∗∗^ *p* < 0.01.

^∗∗∗^ *p* < 0.001.

## 4. Discussion

In the present study, we conducted LPA on ward atmosphere and explored whether physical environment and experience of WPV predicted different profile memberships. Our research revealed the existence of three distinct ward atmosphere profiles. Furthermore, we found that whether there is greenery and video surveillance in the ward, whether smoking is allowed, and the perception of the ward size, tidiness, ventilation, and adequacy of safety measures all have predictive effects on the atmosphere of the ward had differential effects on distinct profiles. Meanwhile, the experience of WPV was also a predictor factor for distinct profiles.

First, we found three distinct profiles of ward atmosphere among psychiatric nurses. The three profiles exhibited similar scoring patterns, with relatively high scores on the dimensions of Therapeutic Hold and Patients’ Cohesion, but lower scores on the Safety Experience dimension. This pattern may be attributed to two factors: Nurses might be reluctant to report negatively on professionally relevant factors like treatment support, while the inherent unpredictability of psychiatric patients naturally leads to lower perceived safety across the board [45, 46]. Moreover, it is noteworthy that the curves for“Safety‐Deficient group” and “Support‐Depleted group” are not nearly parallel but instead exhibit a distinct point of intersection. This finding suggested that ward atmosphere perceptions cannot be simplistically categorized as universally “high,” “medium,” or “low.” Instead, it underscores the importance of identifying the specific underlying psychological characteristics that define each profile.

We proposed that nurses in the “Triple‐High group” generally perceive the ward atmosphere positively. The reason why there are intersections between “Safety‐Deficient group” and “Support‐Depleted group” might be that when patients’ cohesion is high, nurses may become concerned about the potential for collective harmful behaviors, leading to feelings of anxiety and fear [11, 47]. This finding represents a common psychological reaction, and the fact that the “Safety‐Deficient group” including 800 nurses (77.1% of the total sample) further supports the conclusion that safety is a common concern among nurses. Since fear was seen as difficult to manage, it made the nurses actively reflect on their own safety and not come to harm [48]. One of the key roles of nurses in the ward is as managers [49]. It is indicative of nurses’effective management when patients’ cohesion was seriously supported. Interventions should strengthen nurses’ managerial support, safety resources, and institutional protection, rather than relying solely on individual coping.

This study reveals the differential impact of various physical environmental factors on nurses’ perception of ward atmosphere. Ward size constitutes the physical foundation of the psychiatric treatment environment, directly influencing nurses’ work experience and atmosphere perception. Spacious areas effectively reduce conflict risks associated with high density and provide necessary personal space for patients [50], significantly decreasing nurses’ workload in managing patient conflicts [51]. Furthermore, sufficient ward space plays an important role when staff need to divert patient attention or conduct private conversations [50, 52].

Adequate safety measures are also fundamental to the psychiatric treatment environment. Patients’ aggressive behaviors exhibit strong emotional contagion among patients and between patients and nurses, thereby intensifying nurses’ tension [53]. Research indicated that safety measures such as seclusion rooms can reduce incidents of aggression, providing nurses with security and alleviating the psychological and professional burden arising from patient unpredictability [47, 54]. This study further confirmed that “ward size” and “adequate safety measures” show significant differences across all three profiles, indicating that regardless of nurses’ overall perception type, spacious areas and reliable safety measures are universal prerequisites for forming a positive ward atmosphere.

Second, as environmental optimization factors, “prohibition of mobile phone use” and “good ventilation” demonstrate positive effects only among nurses in the “Triple‐High group.” This may be because the perceived value of such factors is based on the fulfillment of spatial needs and basic safety. Only after these needs are met do nurses become more sensitive to order and physical comfort. Allowing patients to use mobile phones raises concerns among nurses about being photographed [55], dissemination of inappropriate information, and the impact of external information on patients with conditions such as anorexia [8]. Good ventilation reduces the concentration of volatile organic compounds, formaldehyde, and carbon dioxide, improving mental state [56]. Relevant studies also showed that improved ventilation conditions are associated with reduced risk of cognitive dysfunction [57] and are linked to enhanced decision‐making ability and productivity in work environments [58]. A study on sick leave and ventilation rates in a Massachusetts manufacturing plant revealed correlations between insufficient ventilation and increased sick leave, health issues, and decreased productivity [59].

Furthermore, factors such as “green plants,” “surveillance facilities,” and “ward tidiness” demonstrated predictive power among nurses in the “Safety‐Deficient group” and “Support‐Depleted group.” This indicated that in environments where nurses’ perception is already relatively negative, such elements serve a “problem‐differentiating” function. For example, surveillance facilities can improve staff and patient safety, monitor self‐harm, violence, and elopement; serve as evidence for investigating incidents and allegations [60]; and protect staff in case of accidents [61]. However, some patients indicate that this may infringe on their privacy rights and exacerbate power imbalances between patients and staff [62]. A Delphi study [63] indicated that 92% of experts thought surveillance facilities are necessary, but there remains significant controversy [64]. The introduction of green plants was said to “prioritize vision,” with users becoming “containers for the eyes, easily traversing outdoor spaces, seemingly bringing psychological benefits” [65]. Beyond adding green plants, biophilic design in architecture can enhance the connection between humans and nature, increasing pleasant moods [19]. However, movable green plants can also be used as weapons by patients, potentially exacerbating nurses’ safety concerns [30]. In hospitals, tidiness is a factor influencing spatial atmosphere; it is not only about aesthetics but also disease prevention [66]. When tidiness is not guaranteed, nurses may doubt their own efficacy.

The experience of WPV was statistically significant only in comparison with the “Triple‐High group” with the other two groups. This suggested that exposure to WPV serves as a decisive disruptive factor distinguishing a positive ward atmosphere from one that is not. Nurses who experience WPV tend to report higher levels of job‐related stress and poorer mental health [67, 68]. As highlighted in a qualitative synthesis, the aftermath of such violence often manifests as a persistent and indelible traumatic experience [69]. This forces nurses into a state of constant hypervigilance at work [70], thereby fundamentally undermining their capacity to perceive the ward atmosphere in a positive light.

Based on the findings of this study, hospital administrators should implement targeted modifications to the physical environment of the wards—an approach that is relatively simple to execute, requires minimal time investment, and can effectively improve the ward atmosphere. In addition, multiple strategies should be adopted to reduce WPV, thereby further enhancing the ward atmosphere.

## 5. Limitations and Future Research

The present study has some limitations. First, our reliance on self‐reported measures is inherently subjective. In the future, objective measurements could be made of the physical environment, such as the specific area of the ward setting. Second, the cross‐sectional design of our study limits our ability to infer causal relationships between ward atmosphere profiles and mental health outcomes. Future research could employ latent transition analysis (LTA), a longitudinal extension of LPA, to explore ward atmosphere profiles over time and their predictors.

## 6. Conclusion

In this study, we used LPA to classify psychiatric nurses’ perceptions of ward atmosphere in closed wards into three distinct profiles: “Triple‐High group,” “Safety‐Deficient group,” and “Support‐Depleted group.” We also examined the associations of these profiles with physical environmental factors and WPV. The findings suggest that healthcare administrators should assess the ward atmosphere as perceived by psychiatric nurses in their clinical settings and fully consider the heterogeneity across different dimensions of ward atmosphere. Targeted interventions—particularly those addressing physical environment features and WPV prevention—should be developed for different profile groups to foster a more positive ward atmosphere and enhance nurse well‐being.

## Author Contributions

Deyu Liu: conceptualization, data curation, investigation, methodology, software, visualization, writing–original draft.

Liping Zhao: resources, writing–review and editing.

Jianjian Wang: conceptualization, funding acquisition, project administration, resources, supervision, writing–review and editing.

## Funding

This work was supported by the Second Xiangya Hospital of Central South University National Institutes of Health (LYG20240084).

## Conflicts of Interest

The authors declare no conflicts of interest.

## Data Availability

The datasets used and/or analyzed during the current study are available from the corresponding author upon reasonable request.
